# Microabrasion: An Effective Method for Improvement of Esthetics in Dentistry

**DOI:** 10.1155/2013/951589

**Published:** 2013-10-08

**Authors:** Biji Balan, Chengappa Madanda Uthaiah, Sreejesh Narayanan, Priyadarshini Mookalamada Monnappa

**Affiliations:** ^1^Department of Conservative Dentistry and Endodontics, Kannur Dental College, Anjarakandy, Kannur, Kerala 670612, India; ^2^Department of Prosthodontics, Kannur Dental College, Anjarakandy, Kannur, Kerala 670612, India; ^3^District HQ Hospital, Kannur, Kerala 670017, India; ^4^Kannur Medical College, Anjarakandy, Kannur, Kerala 670612, India

## Abstract

Enamel microabrasion can eliminate enamel irregularities and discoloration defects, thus improving the appearance of teeth. This paper presents the latest treatment protocol of enamel microabrasion to remove stains on the enamel surface. It has been verified that teeth submitted to microabrasion acquire a yellowish colour because of the thinness of the remaining enamel, revealing the colour of dentinal tissue to a greater degree. Enamel microabrasion is a technique that can be used to correct discoloured enamel. Enamel microabrasion was developed in the mid-1980s as a method of eliminating enamel discolouration defects and improving the appearance of teeth. Several years after the method was developed, much has been learned about this technique, long-term results of treatment, and microscopic changes to the enamel surface that have distinguishable clinical implications. In addition, certain patients can benefit from enamel microabrasion to yield attractive cosmetic results. The aim of this study was to report the clinical case of a male patient of 25 years with moderate fluorosis, whose smile was re-established by the use of an enamel microabrasion technique, with 18% hydrochloric acid and pumice slurry shown to be a safe and efficient method for removing fluorosis stains.

## 1. Introduction

It is well documented that fluoride can have both beneficial and detrimental effects on the dentition ever since Mc Kay and G. V. Black published the effect of fluoride on dentition in 1916 [1]. The beneficial effects of fluoride on dental caries are due primarily to the topical effect of fluoride after the teeth have erupted in the oral cavity. In contrast, detrimental effects are due to systemic absorption during tooth development resulting in dental fluorosis that is one of the most common types of enamel demineralisation [1]. Initially, it involves subsurface hypomineralization and/or porosity of the enamel but may extend through the whole thickness as the severity increases [2]. The discoloration may range from yellow to dark brown [3]. Enamel discoloration especially in the anterior teeth may raise esthetic concerns in patients. Conservative non-restorative methods such as microabrasion technique have been used in the treatment of demineralization defects and discolorations of teeth [4]. This technique which involves mild acid etching in combination with rotary application of an abrasive medium [5] was first described by Dr. Walter Kane (Colorado Springs, 1916). By rubbing six maxillary anterior teeth with hydrochloric acid (HCl) under the flame of an alcohol torch, he found favourable results in the treatment of enamel fluorosis without any destruction or damage of enamel. However, for more than 60 years, most clinicians avoided applying this technique, because of fear of damage or destruction of the enamel [6, 7]. In 1984, McCloskey introduced the use of acid combined with pumice [7] which was named “microabrasion” by Croll two years later [8]. Considering its high incidence, the dentist must be able to diagnose fluorosis properly and treat it efficiently.

If the stains are present in the outer layers of enamel, they can be easily removed, leaving a smooth, glassy enamel surface as the finished result. This surface is seen to be more caries resistant than the original surface. This paper deals with a case report of how effectively mild to moderate stains that are removed using microabrasion technique.

## 2. Case Report-1

**Figure 1:**
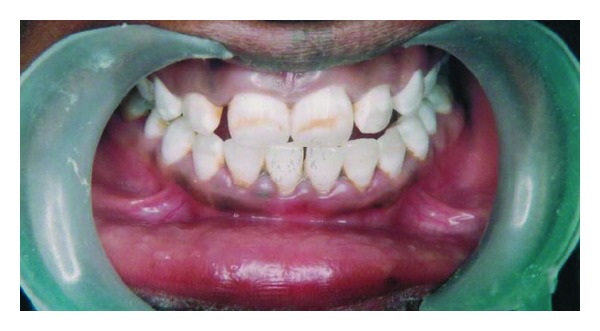
Preoperative.

A twenty-five year-old-male patient came to the dental clinics for routine dental care. His chief complaint was to remove and/or minimize the noticeable brown/yellow staining of his teeth. He wanted the least invasive and most cost-effective treatment to change his smile. A review of his medical history and past dental history revealed no contraindications to dental treatment. In consideration of his age, the patient was not interested in treatment options that involved significant removal of tooth structure, such as porcelain or composite resin veneers. From the appearance of his teeth, a diagnosis of mild fluorosis staining determined by using Dean's Fluorosis Index (Table 1) was sent on the anterior teeth in the aesthetic zone, with the most significant staining occurring on the maxillary anterior teeth, with light brown streaks in the middle third of the facial surfaces (Figure 1).

A treatment plan was presented to the patient that would fulfill his request for minimally invasive treatment which proposed microabrasion of the superficial enamel. Upon completion of treatment, the tooth shade would be evaluated. 

**Figure 2:**
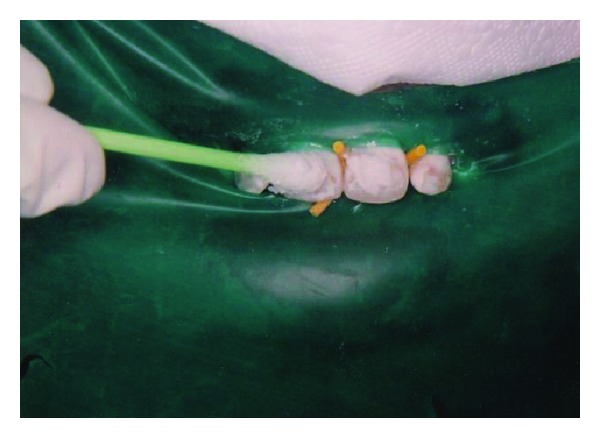
Application of HCL and pumice slurry on teeth.

**Figure 3:**
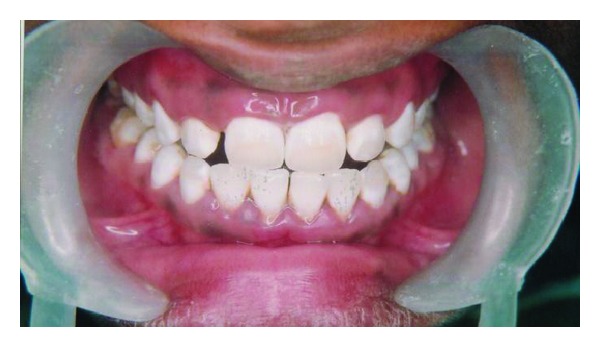
Postoperative.

The teeth was isolated with a rubber dam to protect the gums from coming into contact with the acid (18% HCl). The pumice-acid slurry is then applied to the tooth or teeth to the facial surfaces of the maxillary teeth using cotton (Figure 2) and rubbed either manually or with a very slow speed rubber cup. Using a right angle latch type slow-speed handpiece running the motor at 1,000 rpm, a hybrid bristle brush-cup was used to rub the pumice acid slurry for three separate applications of 30 to 40 seconds each. Between each application, the slurry was rinsed and dried from the tooth surfaces. This procedure was repeated three times. At the completion of the microabrasion technique, the enamel surfaces were polished with a cup-shaped porcelain polishing rubber abrasive to smooth and polish the enamel surface. After a few layers of enamel are removed, the slurry was rinsed with water and the result was evaluated. This process is repeated until the stain is gone or the process must be stopped for other reasons (enamel getting too thin or tooth getting sensitive). After the process was complete, fluoride gel was placed on the teeth in order to reduce postoperative sensitivity. The entire process takes less than an hour and is permanent. The rubber dam was removed and the patient viewed the result of treatment (Figure 3). He was pleased with the result from the immediate removal of the dark staining on his maxillary anterior teeth.

## 3. Case Report-2

**Figure 4:**
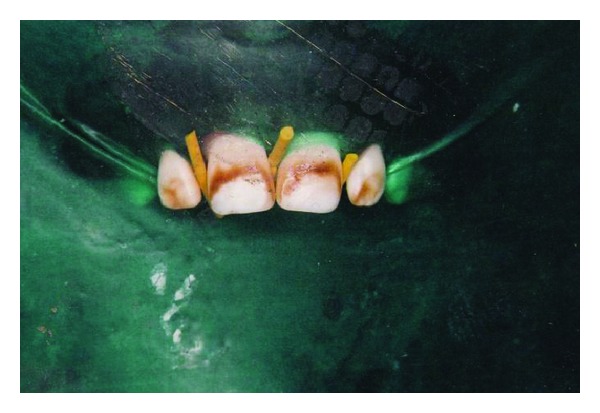
Preoperative. Isolation done using rubber dam.

**Figure 5:**
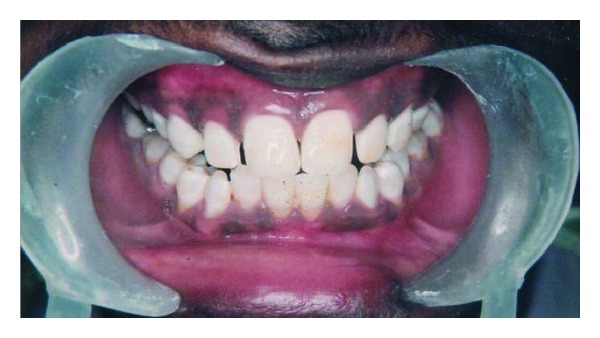
Postoperative.

A thirty-year-old male came to the dental hospital with chief complaint of dark brown staining of the anterior teeth. He also wanted a least invasive, cost-effective treatment to enhance his esthetics. From the appearance of his teeth, diagnosis of moderate flourosis staining was determined by Dean's Fluorosis Index (Table 1). The most significant staining occurring on the maxillary anterior teeth contained dark brown streaks in the middle third of the facial surfaces (Figure 4). The same procedure for the microabrasion was repeated as was done in Section 2. After the procedure was completed, fluoride gel application was placed on the teeth to reduce the postoperative sensitivity. The rubber dam was removed for the evaluation of the result by the patient (Figure 5). The patient was quite satisfied with the results. In the above mentioned cases, patients were asked not to smoke, eat, or drink anything that could possibly stain the teeth for 24–48 hours after the treatment.

## 4. Discussion

Dental fluorosis is defined as hypomineralisation of enamel resulting from excessive intake of fluoride during tooth development. It is characterized by diffuse opacities on the enamel surface. These are differentiated from other conditions by the characteristic bilaterally symmetric distribution of the enamel defects. The degree to which the enamel is affected is dependent upon the duration, timing, and intensity of the fluoride concentration. In its mild form, most commonly the teeth present with small white streaks and the enamel appear mottled. As the severity of the condition increases, black and brown stains develop. The enamel microabrasion technique is an excellent method to remove intrinsic enamel stains of any etiology and colour, as well as to correct superficial irregularities on the buccal aspect of enamel, caused by either amelogenesis imperfecta or defects acquired after removal of orthodontic appliance [9]. These alterations, however, should have a hard texture and may affect the superficial layers of dental enamel. Because it is very difficult to determine the real depth of intrinsic stains or surface irregularities, the application of the microabrasion technique on intrinsic stains, regardless of their dimensions and depths, should always be considered before trying a restorative procedure [10]. Recurrence of staining on the teeth was not seen in these cases after two months on their recall visits. Maintenance of oral hygiene and caries prevention methods was advised to the patients.

Tooth discoloration due to fluorosis is an aesthetic problem for certain patients. While there is a range of restorative interventions that can be used to change the appearance of fluorosed teeth, the goal of minimally invasive treatment for mild-moderate fluorosis is the one that should be evaluated first. For the case presented in this article, a minimally invasive treatment option of microabrasion is the best choice.

## 5. Conclusion

 The current evidence demonstrates that when a diagnosis of fluorosis has been made, the majority of cases are very mild or mild and do not require restorative treatment. For mild fluorosis discoloration and for moderate/severe fluorosis, treatment to change the aesthetic appearance of the teeth can be accomplished with minimally invasive treatment using microabrasion or in case of moderate-severe condition, combinations of microabrasion with bleaching can be done to provide the patient with an aesthetically acceptable results. For more severe fluorosis with dark discolorations and surface pitting, adhesive restorative dentistry may be necessary to fulfill a patient's aesthetic desires.

## Figures and Tables

**Table 1:** 

Normal	The enamel represents the usual translucent semivitriform type of structure. The surface is smooth, glossy, and usually of a pale creamy white colour.

Questionable	The enamel discloses slight aberrations from the translucency of normal enamel, ranging from a few white flecks to occasional white spots. This classification is utilized in those instances where a definite diagnosis of the mildest form of fluorosis is not warranted and a classification of “normal" is not justified.

Very mild	Small, opaque, paper-white areas scattered irregularly over the tooth but not involving as much as 25% of the tooth surface. Frequently included in this classification are teeth showing no more than about one to 2 mm of white opacity at the tip of the summit of the cusps of the bicuspids or second molars.

Mild	The white opaque areas in the enamel of the teeth are more extensive but do not involve as much as 50% of the tooth.

Moderate	All enamel surfaces of the teeth are affected, and the surfaces subject to attrition show wear. Brown stain is frequently a disfiguring feature

Severe	Includes teeth formerly classified as “moderately severe and severe.” All enamel surfaces are affected and hypoplasia is so marked that the general form of the tooth may be affected. The major diagnostic sign of this classification is discrete or confluent pitting. Brown stains are widespread and teeth often present a corroded-like appearance.
